# Luspatercept mitigates bone loss driven by myelodysplastic neoplasms and estrogen-deficiency in mice

**DOI:** 10.1038/s41375-022-01702-1

**Published:** 2022-09-29

**Authors:** Heike Weidner, Manja Wobus, Lorenz C. Hofbauer, Martina Rauner, Uwe Platzbecker

**Affiliations:** 1 Department of Medicine III and Center for Healthy Aging, Technische Universität Dresden, Dresden, Germany; 2 Department of Medicine I, Technische Universität Dresden, Dresden, Germany; 3 German Cancer Consortium (DKTK), partner site Dresden and German Cancer Research Center (DKFZ), Heidelberg, Germany; 4 Medical Clinic and Policlinic I, Hematology and Cellular Therapy, University Hospital Leipzig, Leipzig, Germany

**Keywords:** Cell biology, Anaemia

## To the Editor:

Transforming growth factor (TGF)-beta signaling is involved in the proliferation and differentiation of cells within the osteo-hematopoietic niche. The TGF-beta subfamily members activin A and B, as well as growth and differentiation factor (GDF)-8 and GDF-11 bind to the activin receptor type 2A/B (ACVR2A and ACVR2B) and phosphorylate the type I receptor ALK-4/-5/-7 for activation of SMAD2/3. The bone morphogenetic proteins (BMP), on the other hand, induce SMAD1/5/8 signaling by binding to the type II receptor BMPR2 and form a complex with ALK-2/-3/-6 [1]. In particular BMP-4/6 have been shown to preferentially bind to ACVR2B to activate the SMAD1/5/8 signaling [2]. Both SMAD complexes bind to SMAD4, which allows the translocation into the nucleus to regulate gene expression [1]. Dysregulation of this pathway caused by deficiency or overexpression can occur in hematological diseases including beta-thalassemia and myelodysplastic neoplasms (MDS), which are both associated with altered bone metabolism leading to bone loss and lower bone strength [3–5]. Luspatercept, which has been approved for the treatment of MDS and beta-thalassemia, consists of the extracellular domain of the ACVR2B and IgG2a-Fc domain to trap TGF-β superfamily ligand [3]. Its advanced ligand selectivity with a high affinity to GDF-8/-11 combined with low affinity to activin B, and no binding to activin A contribute to its effects to enhance erythroid maturation [6]. In patients with MDS, GDF-11, which inhibits late-stage differentiation of erythroid precursors, correlates negatively with hemoglobin and red blood cell (RBC) levels [7]. However, the hematopoietic-specific *Gdf11* knockout in MDS mice does not affect RBC [8]. In addition, endogenous SMAD2 phosphorylation is intrinsically active in MDS due to the reduced inhibitory SMAD7 levels [3]. Luspatercept has been demonstrated to improve RBC maturation in murine models and patients with MDS as well as beta-thalassemia [5, 6, 9, 10]. Besides ineffective hematopoiesis, many patients with MDS have an osteoporotic bone phenotype beyond age-related causality [4]. Recently, we reported that exposure of MSC to luspatercept modulates their hematopoietic support in vitro by increasing HSC differentiation potential, homing and engraftment [11]. It is also known, that postmenopausal women show elevated GDF-11 levels, which correlates negatively with bone mass [12], and blocking GDF-11 signaling prevents estrogen deficiency-induced bone loss in mice [13]. The effects of RAP-536, the murine version of luspatercept, on bone were so far only analyzed in beta-thalassemia showing an increased bone mass in thalassemic but not in control mice [5].

**Fig. 1:**
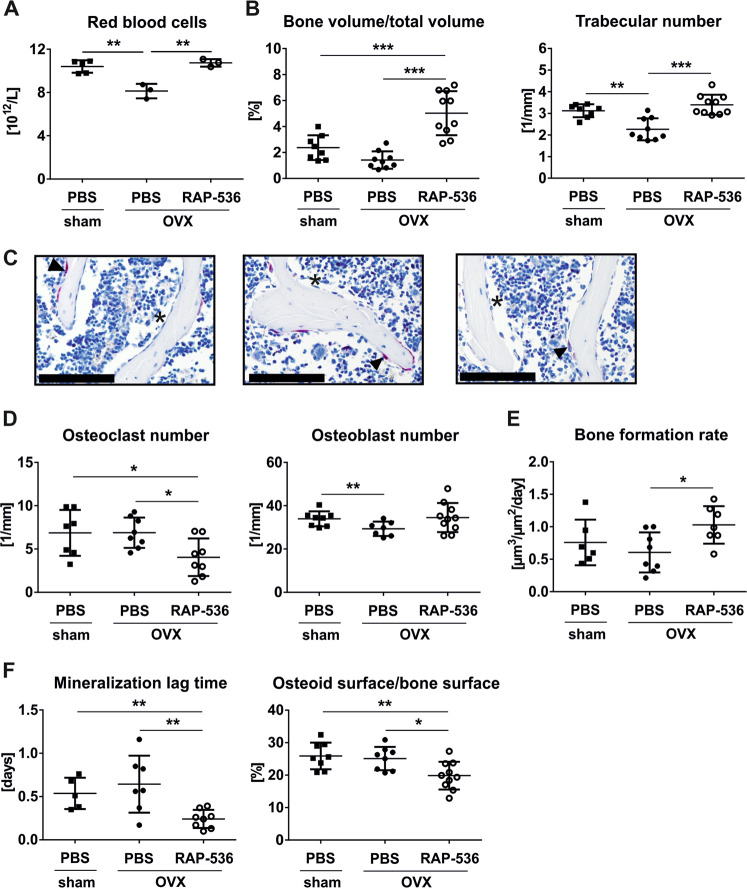
RAP-536 prevents anemia and bone loss in estrogen-deficient mice. Ten-week-old female wild-type mice were divided into three groups: sham, bilaterally ovariectomized (OVX), and OVX + RAP-536. RAP-536 administration (10 mg/kg, intraperitoneally twice per week for 4 weeks) directly starts after OVX and the other mice received PBS as control. After treatment, **A** red blood cells (sham: *n* = 5; OVX: *n* = 3; OVX + RAP-536: *n* = 3) were analyzed with the Sysmex XN-1000 (Sysmex, Norderstedt, Germany) and **B** the bone volume per total volume (sham: *n* = 8; OVX: *n* = 9; OVX + RAP-536: *n* = 10) as well as the trabecular number (sham: *n* = 8; OVX: *n* = 9; OVX + RAP-536: *n* = 10) of femora were assessed using micro-computed tomography (vivaCT40, SCANCO Medical, Brüttisellen, Switzerland). Representative images of tartrate-resistant acid phosphatase (TRAP)-stained femora are depicted in **C** using the CellSens program and Microscope Axio Imager M1 (Carl Zeiss Jena, Jena, Germany). Arrows heads indicate osteoclasts and asterisks osteoblasts. Original magnification ×20 (scale bars = 100 μm). Quantitative data of **D** osteoclasts (sham: *n* = 7; OVX: *n* = 8; OVX + RAP-536: *n* = 8) and osteoblasts per bone perimeter (sham: *n* = 8; OVX: *n* = 7; OVX + RAP-536: *n* = 10) in TRAP-stained femora. **E** Five and 2 days before sacrifice, mice received intraperitoneal calcein injections. To assess the bone formation rate in vertebrae (sham: *n* = 6; OVX: *n* = 8; OVX + RAP-536: *n* = 7), double labeling was analyzed. **F** Vertebrae were used for von Kossa/van Gieson staining to determine the mineralization lag time (sham: *n* = 5; OVX: *n* = 7; OVX + RAP-536: *n* = 8) and the osteoid surface per bone surface (sham: *n* = 8; OVX: *n* = 8; OVX + RAP-536: *n* = 10). Data are shown as mean ± SD of one experiment (**A**) or three independent experiments (**B**–**F**). Statistical analysis was performed by one-way ANOVA followed by Bonferroni´s comparison. *p < 0.05; **p < 0.01; ***p < 0.001.

Here, we report the effects of RAP-536 on bone health in wild-type, estrogen-deficient, and MDS mice. First, 10-week-old female wild-type C57BL/6J (WT) mice were treated 3 weeks with 10 mg/kg RAP-536 (provided by BMS/Acceleron, Cambridge, MA, USA) or PBS as control. As expected, RAP-536 treatment increased the number of RBC. Furthermore, the bone volume and trabecular number were elevated by 70% (*p* < 0.01) and 20% (*p* < 0.05), respectively, in RAP-536-treated compared to control mice. This increase was due to a dual mode of action, including reduced osteoclast-covered bone surface (−25%; *p* < 0.05) concomitant with a normal amount of osteoblasts. Despite similar numbers of osteoblasts, RAP-536 treatment enhanced the bone formation rate (BFR) (2.4-fold; *p* < 0.001), which was associated with a lower osteoid maturation time (−33%; *p* < 0.05) (Supplementary Table 1). Thus, RAP-536 has both erythroid-stimulating and osteo-anabolic effects. To analyze whether RAP-536 also prevents bone loss in estrogen-deficient mice, we used 10-week-old female WT mice and divided them into three groups: sham, bilaterally ovariectomized (OVX), and OVX + RAP-536. The mice of the latter group were treated with 10 mg/kg RAP-536 for 4 weeks starting on the day of OVX. Simultaneously, all mice in the sham and OVX group received PBS. After OVX, estrogen-deficient mice developed anemia, which was prevented by concurrent RAP-536 treatment (Fig. 1A and Supplementary Fig. 1A). As expected, OVX resulted in trabecular (−28%; *p* < 0.01) and cortical femoral bone loss (−9%; *p* < 0.01) (Fig. 1B and Supplementary Fig. 1B). Similar to the RAP-536-treated WT mice, RAP-536 increased bone volume 3.6-fold (*p* < 0.001) and the trabecular number by 50% (*p* < 0.001) in OVX-treated mice caused by a reduced number of osteoclasts (−41%, *p* < 0.05) and an increased osteoblast activity measured by procollagen type 1 N-terminal propeptide (+50%; *p* < 0.05) together with an elevated mineral apposition rate (+94%; *p* < 0.01) and higher BFR (+93%; *p* < 0.05) (Fig. 1C–E and Supplementary Fig. 1C, D). Despite the reduced osteoblast number after OVX, mineralization parameters were not affected. RAP-536 treatment of OVX mice nevertheless resulted in faster mineralization, which was reflected in lower osteoid surface (−21%; *p* < 0.05) and width (−20%; *p* < 0.001) (Fig. 1F and Supplementary Fig. 1E).

**Fig. 2:**
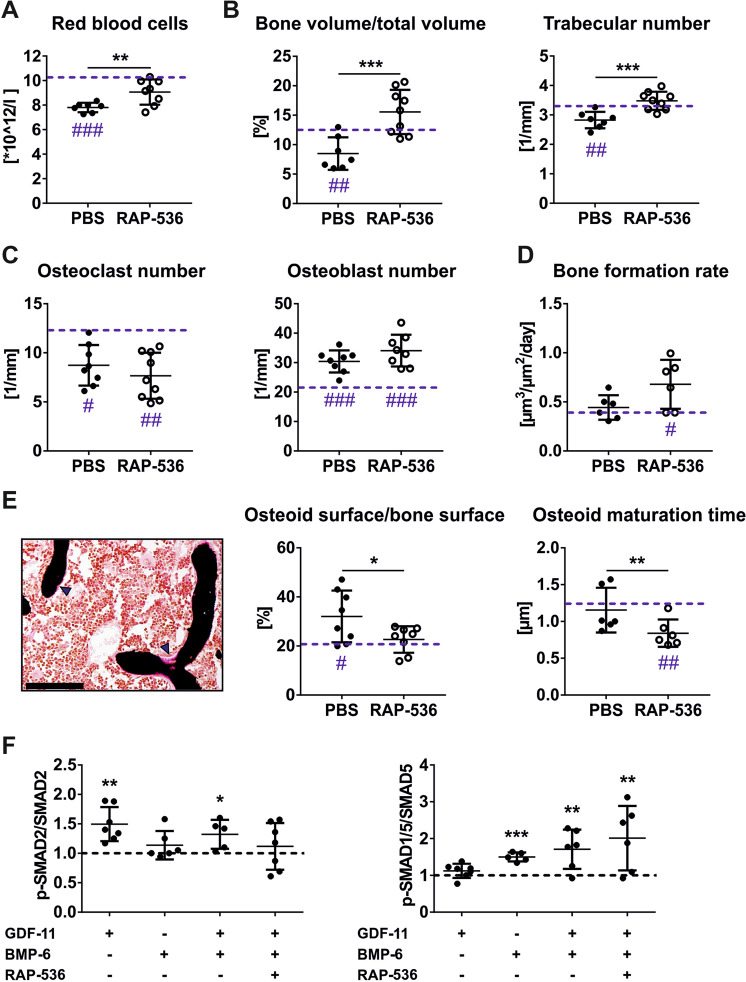
RAP-536 abolishes anemia and bone loss in MDS mice. **A** After 3-weeks of PBS or RAP-536 treatment (15 mg/kg, intraperitoneally twice per week), the number of red blood cells (PBS: *n* = 7; RAP-536: *n* = 8) was analyzed by Sysmex XN-1000 (Sysmex, Norderstedt, Germany) in 6-month-old female NUP98/HOXD13 (NHD13) mice. **B** Using micro-computed tomography (vivaCT40, SCANCO Medical, Brüttisellen, Switzerland) the bone volume per total volume (PBS: *n* = 7; RAP-536: *n* = 9) and trabecular number (PBS: *n* = 7; RAP-536: *n* = 9) were assessed in vertebrae. **C** To quantify the osteoclast (PBS: *n* = 8; RAP-536: *n* = 9) and osteoblast number (PBS: *n* = 8; RAP-536: *n* = 8), vertebrae were stained with tartrate-resistant acid phosphatase. **D** Double calcein labeling was used to determine bone formation rate (PBS: *n* = 6; RAP-536: *n* = 6). **E** Left: representative image of von Kossa/van Gieson stained vertebra are depicted by CellSens program and Microscope Axio Imager M1 (Carl Zeiss Jena, Jena, Germany). Osteoid is stained in pink and indicated by arrowheads. Original magnification ×20 (scale bars = 100 µm). Right: quantification of osteoid surface per bone surface (PBS: *n* = 8; RAP-536: *n* = 8) as well as osteoid maturation time (PBS: *n* = 6; RAP-536: *n* = 6). The dotted line represents aged-matched wild-type (WT) levels. Data are shown as mean ± SD. Statistical analysis was performed by the two-sided Student’s *t* test. *p < 0.05; **p < 0.01; ***p < 0.001 vs. NHD13 PBS. ^#^*p* < 0.05; ^##^*p* < 0.01; ^###^*p* < 0.001 vs. WT PBS. **F** In vitro differentiated WT osteoblast (day 10) were treated with 100 ng/ml GDF-11, 100 ng/ml BMP-6 and/or 10 µg/ml RAP-536 for 30 min. Ser465/467-phosphorylation of SMAD2 and Ser463/465-phosphorylation of SMAD1/5 were evaluated using Western blotting and normalized to SMAD2 and SMAD5, respectively, beta-actin, as well as untreated WT osteoblasts. *n* = 5–8. The dotted line represents untreated WT osteoblasts. Data are shown as mean ± SD of three independent experiments. Statistical analysis was performed by the two-sided Student’s *t* test.

Based on the positive bone outcome of RAP-536 in WT mice, we tested the effects of RAP-536 on bone in a preclinical model of MDS. Similar to male C57BL/6-Tg(Vav1-NUP98/HOXD13)G2Apla/J (NHD13) mice [4, 14], female mice also have a permanent altered bone formation from 8 to 24 weeks of age (Fig. 2 and Supplementary Fig. 2). Thus, the onset of the MDS-like hematological phenotype is with 16 weeks of age and in the next 8 weeks the severity of anemia increases, 21-week-old female NHD13 and their littermate WT mice were treated 3 weeks with 15 mg/kg RAP-536 or PBS. Since NHD13 mice have established anemia and a reduced bone volume, we used a higher RAP-536 dose in this model. As expected, RAP-536 improved erythropoiesis but had no effect on white blood cells and platelets (Fig. 2A and Supplementary Fig. 3A). Similar to 6-month-old male NHD13 mice [14], bone loss in vertebrae of female NHD13 mice is caused by an insufficient bone mineralization with high numbers of osteoblasts and low numbers of osteoclasts. RAP-536 treatment restored bone volume by increasing the trabecular number (+23%; *p* < 0.001), but not the trabecular thickness, leading to higher stiffness of vertebrae (2.2-fold; *p* < 0.01) (Fig. 2B and Supplementary Fig. 3B, C). However, in contrast to WT mice, RAP-536 did not reduce the osteoclast number or their activity in NHD13 mice. Instead, the positive effect on bone metabolism was induced by promoting osteoblast maturation. In MDS mice, RAP-536 did not further induce bone formation or increase osteoblast number, but stimulated existing osteoblasts to mineralize more rapidly (Fig. 2C–E and Supplementary Fig. 3D–F).

Finally, we analyzed the aspect that ACVR2B also has a high affinity for BMP-4/6 and stimulates osteoblastogenesis via SMAD1/5/8 [2] in WT osteoblasts in vitro. GDF-11 but not BMP-6 induced SMAD2 phosphorylation, which was suppressed by RAP-536. In contrast, only BMP-6 stimulated SMAD1/5 phosphorylation and after GDF-11 was trapped by RAP-536 the phospho-SMAD1/5/SMAD5 ratio was even higher (Fig. 2F). Therefore, RAP-536 allows for SMAD1/5/8 signaling in osteoblasts, which may suggest osteoblast-promoting effects.

In this study, RAP-536 stimulated erythropoiesis in all models, and the direct effect was confirmed by an increased colony number using erythroid burst-forming unit assay (Supplementary Fig. 3). Of note, the inhibition of more than one ACVR2B ligand seems to be required to increase RBC. Since erythroid cells have a low *Acvr2b* expression, indirect influences should be considered because bone-forming osteoblasts also express this receptor. While blocking GFD-8 and GDF-11 may account for the decreased osteoclast numbers, only GDF-11 antibodies increase bone formation in ovariectomized mice [13, 15]. Here, we demonstrated that the bone gain after RAP-536 treatment in estrogen-deficient and NHD13 mice can be attributed to the reduction of bone resorption and/or stimulation of osteoblast maturation. Thus, RAP-536 represents promising strategy to prevent bone loss in two mouse models that fundamentally differ in their pathogenesis. The regulation of bone homeostasis opens the possibility that RAP-536 indirectly stimulates erythropoiesis through bone cells, which reside within the osteo-hematopoietic niche. Taken together, luspatercept not only improves erythropoiesis in WT, OVX, and MDS mice but also their bone phenotype. Therefore, this aspect may be beneficial for diseases like MDS in which anemia and bone loss coincide. Studies may also be warranted in patients with postmenopausal osteoporosis and concomitant anemia.

## Supplementary information

Supplemental information clean

Supplemental Figures

Supplementary Table 1

## Data Availability

The described methods and generated data of the current study are available from the corresponding author upon request.
