# Supplementary material for: Luspatercept mitigates bone loss driven by myelodysplastic neoplasms and estrogen-deficiency in mice
Source: Leukemia. 2022 Sep 29;36(11):2715–8. doi: 10.1038/s41375-022-01702-1 (PMC9613459; doi:10.1038/s41375-022-01702-1)
Supplement: Supplementary file 1 — Supplemental information clean [file 41375_2022_1702_MOESM1_ESM.docx]

**Supplemental data: Methods**

**Treatment of C57BL/6J, NHD13, and littermate control mice**

All animals were housed under institutional guidelines in the animal facility of Technische Universität Dresden including feeding a standard diet as well as water *ad libitum*, and exposure to 12 h light/dark cycle in an air-conditioned room at 23°C. The institutional animal care committee and the Federal State of Saxony approved all procedures (TVV 14/2017 and TVV 48/2016). All mice were treated intraperitoneally twice per week with RAP-536 or PBS as control. To assess bone formation, all mice received intraperitoneal calcein injections (20 mg/kg) five and two days before sacrifice. At baseline and on the day of sacrifice, hematological parameters were analyzed in peripheral blood samples. Only NHD13 mice with an anemia at the beginning of the experiment were included in the study. On the day of sacrifice, serum for ELISA, long bones, as well as spine were collected for molecular and histomorphometric analyses. To analyze the bone microarchitecture, the femora of PBS- or RAP-536-treated WT mice were used for micro-computed tomography. All analyses were performed blindly for two experimentators.

**Supplemental data: Figures**

**Supplementary Fig. 1: RAP-536 effects on erythropoiesis and bone metabolism of estrogen-deficient mice.**

Ten-week-old female wild-type mice were divided into 3 groups: sham, bilaterally ovariectomized (OVX), and OVX + RAP-536. RAP-536 administration (10 mg/kg, intraperitoneally twice per week for 4 weeks) directly starts after OVX and the other mice received PBS as control. After treatment, (A) hemoglobin (sham: n=5; OVX: n=3; OVX+RAP-536: n=3) and hematocrit (sham: n=5; OVX: n=3; OVX+RAP-536: n=3) were analyzed with the Sysmex XN-1000 (Sysmex, Norderstedt, Germany). (B) The cortical thickness of femora (sham: n=8; OVX: n=9; OVX+RAP-536: n=10) were assessed using micro-computed tomography (vivaCT40, SCANCO Medical, Brüttisellen, Switzerland). (C) Bone turnover marker procollagen type 1 N-terminal propeptide (P1NP) was measured by ELISA (sham: n=8; OVX: n=5; OVX+RAP-536: n=6). (D) Double calcein labeling was used to determine mineral apposition rate in vertebrae (sham: n=6; OVX: n=8; OVX+RAP-536: n=8). In addition, vertebrae slices were stained with von Kossa/van Gieson to evaluate the osteoid maturation time (sham: n=5; OVX: n=8; OVX+RAP-536: n=8) and osteoid width (sham: n=8; OVX: n=9; OVX+RAP-536: n=10). Data are shown as mean ± SD of one experiment (A) or three independent experiments (B-D). Statistical analysis was performed by the two-sided Student´s t-test. **P*<0.05; ***P*<0.01; ****P*<0.001.

**Supplementary Fig. 2: Impaired bone formation in 8-week-old NHD13 mice.**

Eight-week-old female NUP98/HOXD13 (NHD13) mice were used for the quantitative analysis of (A) the osteoblasts number after tartrate-resistant acid phosphatase staining (WT: n=7; NHD13: n=5), (B) bone formation rate using the double calcein labeling (WT: n=7; NHD13: n=6), and (C) the osteoid surface per bone surface after von Kossa/van Gieson staining (WT: n=5; NHD13: n=6). Data are shown as mean ± SD of three independent experiments. Statistical analysis was performed by the two-sided Student´s *t*-test. **P*<0.05.

**Supplementary Fig. 3: RAP-536 effects on hematopoiesis and bone metabolism in MDS mice.**

(A) After 4-weeks of PBS or RAP-536 treatment (15 mg/kg, intraperitoneally twice per week), the blood parameters (hemoglobin: PBS: n=7; RAP-536: n=7; white blood cells: PBS: n=8; RAP-536: n=9; platelets: PBS: n=8; RAP-536: n=9) were analyzed by Sysmex XN-1000 (Sysmex, Norderstedt, Germany) in 6-month-old female NUP98/HOXD13 (NHD13) mice. (B) Using micro-computed tomography (vivaCT40, SCANCO Medical, Brüttisellen, Switzerland) the trabecular thickness (PBS: n=7; RAP-536: n=9) and separation (PBS: n=7; RAP-536: n=9) were assessed in vertebrae. (C) The stiffness of vertebrae was measured using compression testing (PBS: n=6; RAP-536: n=7). (D) Bone turnover marker C-terminal telopeptide of type 1 collagen (CTX-I) (PBS: n=8; RAP-536: n=7) and procollagen type 1 N-terminal propeptide (P1NP) (PBS: n=6; RAP-536: n=8) were measured by ELISA. (E) To assess the mineral surface (PBS: n=6; RAP-536: n=6) and mineral apposition rate (PBS: n=6; RAP-536: n=5) in vertebrae, double labeling was analyzed. (F) Quantitative data of osteoid width (PBS: n=8; RAP-536: n=10) and mineralization lag time (PBS: n=4; RAP-536: n=6) in von Kossa/van Gieson-stained vertebrae. The dotted line represents aged-matched wild-type (WT) levels. Data are shown as mean ± SD of three independent experiments. Statistical analysis was performed by the two-sided Student´s *t*-test. **P*<0.05; ***P*<0.01; ****P*<0.001 vs. NHD13 PBS. ^#^*P*<0.05; ^##^*P*<0.01; ^###^*P*<0.001 vs. WT PBS.

**Supplementary Fig. 4. RAP-536 increases the number of erythroid colonies.**

Bone marrow cells of wild-type (WT) mice were used for the erythroid burst-forming unit assay. Erythroid colonies were counted after 8 days of cultivation. PBS: n=9, RAP-536: n=5. Data are shown as mean ± SD of two independent experiments. Statistical analysis was performed by the two-sided Student´s *t*-test. ****P*<0.001 vs. PBS.
