# Supplementary figures and images for: Luspatercept mitigates bone loss driven by myelodysplastic neoplasms and estrogen-deficiency in mice
Source: Leukemia. 2022 Sep 29;36(11):2715–8. doi: 10.1038/s41375-022-01702-1 (PMC9613459; doi:10.1038/s41375-022-01702-1)

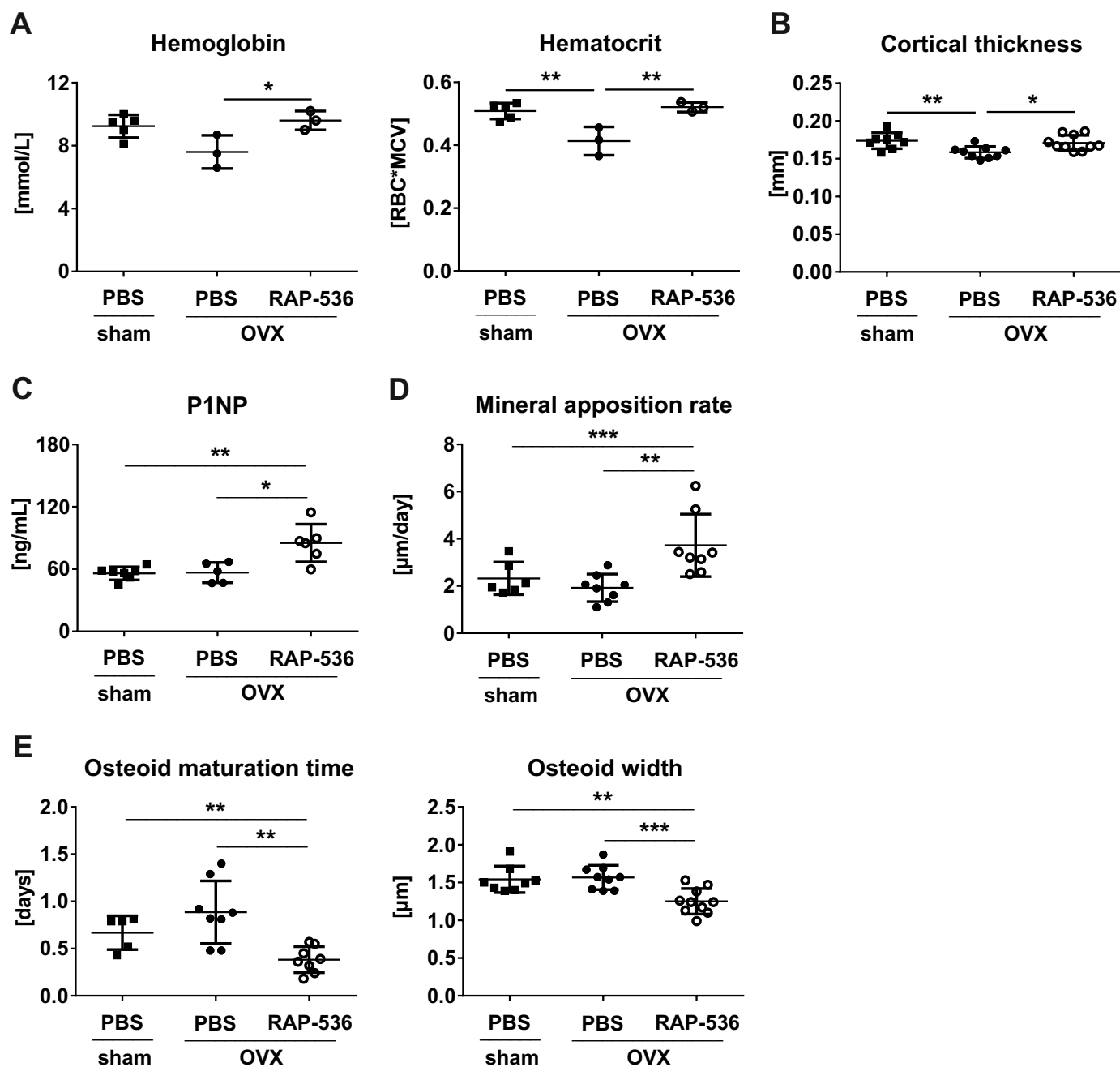

Supplementary Fig. 1

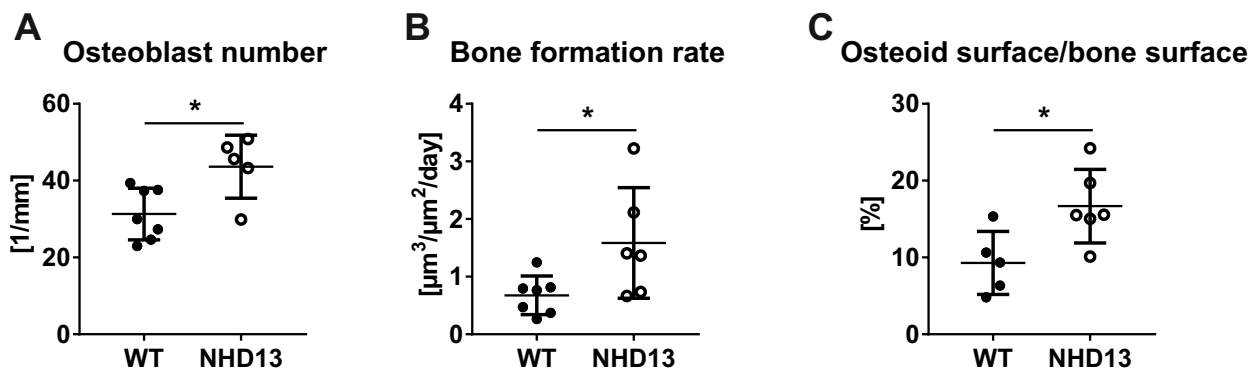

**Supplementary Fig. 2**

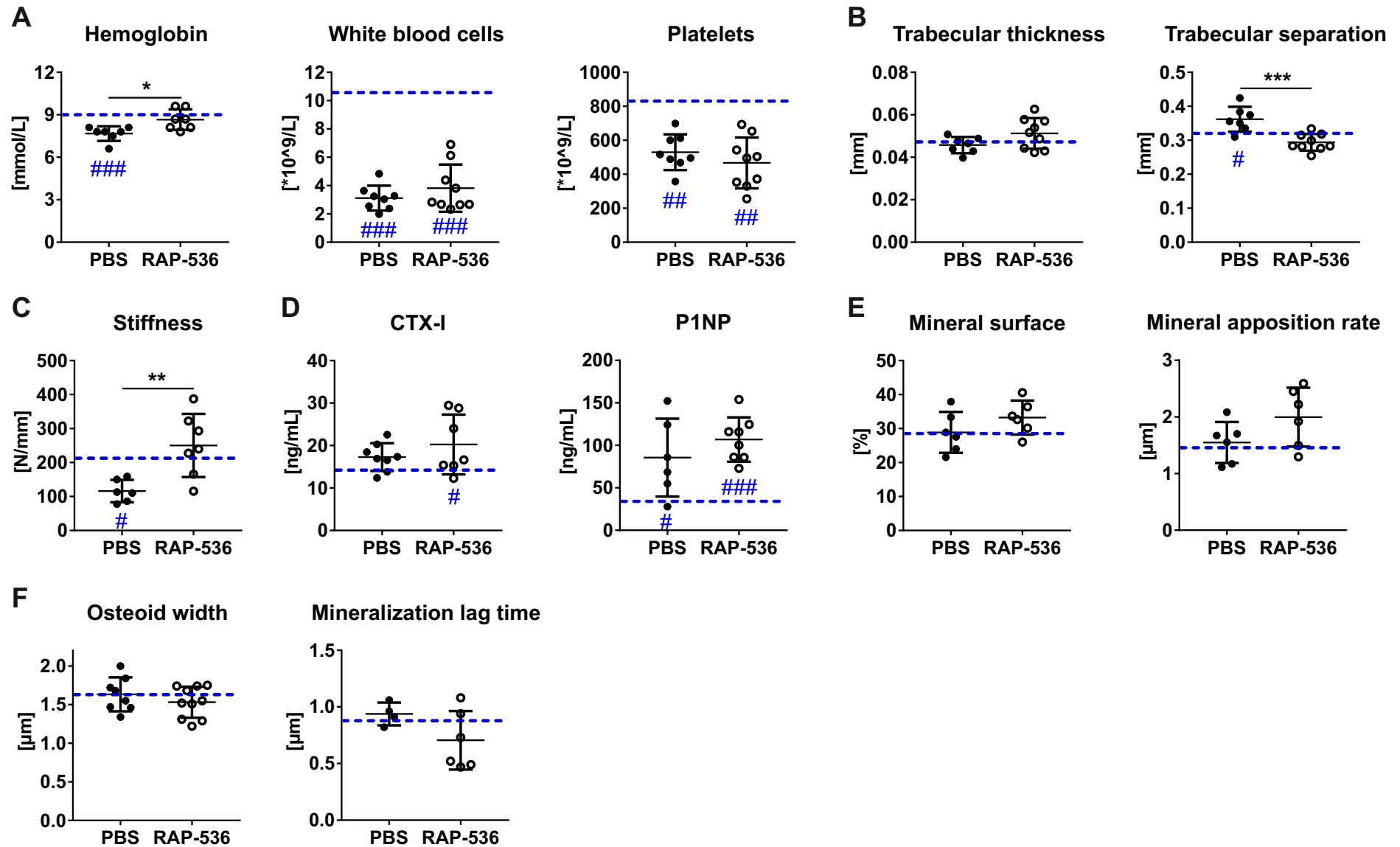

Supplementary Fig. 3

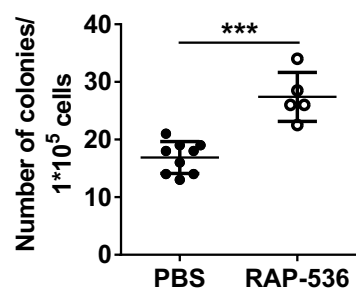

**Supplementary Fig. 4**

Supplement: Supplementary file 2 — Supplemental Figures [file 41375_2022_1702_MOESM2_ESM.pdf]
