# Supplementary material for: Luspatercept mitigates bone loss driven by myelodysplastic neoplasms and estrogen-deficiency in mice
Source: Leukemia. 2022 Sep 29;36(11):2715–8. doi: 10.1038/s41375-022-01702-1 (PMC9613459; doi:10.1038/s41375-022-01702-1)
Supplement: Supplementary file 3 — Supplementary Table 1 [file 41375_2022_1702_MOESM3_ESM.docx]

**Supplemental data: Table**

**Table S1. Bone and serum parameters of wild-type mice after 3 weeks of RAP-536 treatment.**

|  | **Control** | **RAP-536** | ***P* value** |
| --- | --- | --- | --- |
| **Blood parameter** | | | |
| **Red blood cells [10^12^/l]** | 10.2 ± 0.92 | 11.8 ± 0.82 | < 0.010 |
| **µCT parameters of femora** | | | |
| **Bone volume/total volume [%]** | 3.14 ± 1.48 | 5.47 ± 1.62 | < 0.010 |
| **Trabecular number [1/mm]** | 3.06 ± 0.49 | 3.66 ± 0.54 | < 0.050 |
| **Trabecular thickness [µm]** | 41.1 ± 3.00 | 41.3 ± 1.70 | 0.859 |
| **Trabecular separation [mm]** | 0.34 ± 0.05 | 0.27 ± 0.03 | < 0.010 |
| **Cortical thickness [mm]** | 0.20 ± 0.02 | 0.19 ± 0.01 | 0.271 |
| **Histological parameters of vertebrae** | | | |
| **Osteoclast surface [%]** | 11.3 ± 2.5 | 8.5 ± 2.1 | < 0.050 |
| **Osteoblast number [1/mm]** | 14.3 ± 7.06 | 13.9 ± 5.90 | 0.900 |
| **Bone formation rate [µm^3^/µm^2^/day]** | 0.38 ± 0.11 | 0.90 ± 0.16 | < 0.001 |
| **Mineral surface [%]** | 18.8 ± 2.1 | 30.3 ± 3.8 | < 0.001 |
| **Mineral apposition rate [µm/day]** | 1.85 ± 0.24 | 3.05 ± 0.61 | < 0.010 |
| **Osteoid surface/bone surface [%]** | 22.4 ± 6.8 | 24.3 ± 5.3 | 0.551 |
| **Osteoid width [µm]** | 1.65 ± 0.11 | 1.45 ± 0.13 | < 0.010 |
| **Mineralization lag time [days]** | 0.53 ± 0.12 | 0.35 ± 0.14 | < 0.050 |
| **Osteoid maturation time [days]** | 0.67 ± 0.20 | 0.45 ± 0.12 | < 0.050 |
| **Serum parameters** | | | |
| **CTX-I [ng/ml]** | 16.5 ± 2.8 | 23.3 ± 5.5 | < 0.050 |
| **P1NP [ng/ml]** | 54.3 ± 9.9 | 50.7 ± 8.0 | 0.473 |

CTX‑1, C-terminal telopeptide of type 1 collagen; P1NP, procollagen type 1 N-terminal propeptide; Control and RAP-536 (n=6‑10 mice/group). Data are shown as mean ± SD and were analyzed two-sided Student´s *t*-test.
